# Machine Learning Algorithm for Efficient Design of Separated Buffer Super-Junction IGBT

**DOI:** 10.3390/mi14020334

**Published:** 2023-01-28

**Authors:** Ki Yeong Kim, Tae Hyun Hwang, Young Suh Song, Hyunwoo Kim, Jang Hyun Kim

**Affiliations:** 1 Department of Electrical Engineering, Pukyong National University, Busan 48513, Republic of Korea; 2 Department of Electrical and Computer Engineering, Seoul National University, Seoul 08826, Republic of Korea; 3 School of Electronic and Electrical Engineering, Hankyong National University, Anseong 17579, Republic of Korea

**Keywords:** power semiconductor, insulated gate bipolar transistor (IGBT), super-junction IGBT (SJBT), trade-off, turn-off loss (*E*_off_), on-state voltage (*V*_on_), breakdown voltage (BV), machine learning (ML), neural network (NN), optimization, reverse engineering

## Abstract

An improved structure for an Insulated Gate Bipolar Transistor (IGBT) with a separated buffer layer is presented in order to improve the trade-off between the turn-off loss (*E*_off_) and on-state voltage (*V*_on_). However, it is difficult to set efficient parameters due to the increase in the new buffer doping concentration variable. Therefore, a machine learning (ML) algorithm is proposed as a solution. Compared to the conventional Technology Computer-Aided Design (TCAD) simulation tool, it is demonstrated that incorporating the ML algorithm into the device analysis could make it possible to achieve high accuracy and significantly shorten the simulation time. Specifically, utilizing the ML algorithm could achieve coefficients of determination (*R*^2^) of *V*_on_ and *E*_off_ of 0.995 and 0.968, respectively. In addition, it enables the optimized design to fit the target characteristics. In this study, the structure proposed for the trade-off improvement was targeted to obtain the minimum *E*_off_ at the same *V*_on_, especially by adjusting the concentration of the separated buffer. We could improve *E*_off_ by 36.2% by optimizing the structure, which was expected to be improved by 24.7% using the ML approach. In another way, it is possible to inversely design four types of structures with characteristics close to the target characteristics (*E*_off_ = 1.64 μJ, *V*_on_ = 1.38 V). The proposed method of incorporating machine learning into device analysis is expected to be very strategic, especially for power electronics analysis (where the transistor size is comparatively large and requires significant computation). In summary, we improved the trade-off using a separated buffer, and ML enabled optimization and a more precise design, as well as reverse engineering.

## 1. Introduction

Recently, along with the emerging self-driving car market, there has been a growing need for electric vehicles with high power conversion efficiency. According to this recent industrial demand, the steady development of power semiconductors has been utilized for electric vehicles, especially in converters of uninterruptible power supply (UPS) [1,2,3]. For designing power semiconductors, the Insulated Gate Bipolar Transistor (IGBT) [4,5,6] is a power switching device that basically operates like a BJT, showing a low forward voltage drop characteristic, and can withstand a high breakdown voltage (BV) owing to the low concentration of the n-drift region [7,8,9,10]. For this reason, IGBTs are widely used as power semiconductors, and super-junction IGBTs (SJBTs) have an especially high BV caused by fully depleted pillars [11]. Despite these advantages, the injected holes in the p-pillar in the turn-off state become a problem in that they increase the turn-off loss (*E*_off_) [12,13]. As soon as the device is turned off, the holes in the p-pillar cannot be extracted immediately, causing loss. To solve this problem, a dual-gate SJ-IGBT (DG-IGBT) for unipolar turn-off [14] and an IGBT with a depletion trench (DT-IGBT) for active electron extraction [15] have been proposed. However, these devices have a trade-off between *V*_on_ and *E*_off_. In a previous report, we propose an SJBT with separated n-buffer layers to solve the relatively long time required for carrier annihilation during turn-off. However, numerous combinations of possible buffer concentrations make it inefficient to simulate by setting arbitrary concentrations. Moreover, optimization takes longer as the structure becomes more complex, and more models are used [16].

In this study, we investigated a separated buffer SJBT (SB-SJBT) for controlling holes in pillars. The SB-SJBT contains buffers with different concentrations, which improves the trade-off by improving *E*_off_ based on the same *V*_on_. In general, *E*_off_ decreases as the concentration of the p-side n-buffer (p-buffer) increases but adversely affects BV and *V*_on_; therefore, it is necessary to compensate for *V*_on_ with an appropriate concentration combination by lowering the concentration of the n-side n-buffer (n-buffer). Thus, we propose a new approach using a machine learning (ML) algorithm. This approach rapidly and reliably predicts and optimizes the device characteristics [17]. Additionally, it is useful for controlling variables. For instance, when a designer tries to increase the BV of a device, it is made clear which of the parameters, such as device length and drift region concentration, should be adjusted. The remainder of this paper is organized as follows: The SJBT structure and characteristics and the ML algorithm are explained in Section II. Section III shows the verification of the algorithm for its reliability and its application for improving the performance and reverse engineering the parameters of the device with the targeted characteristics.

## 2. Structure and Methodology

### 2.1. Proposed Structure and Characteristics

**Figure 1:**
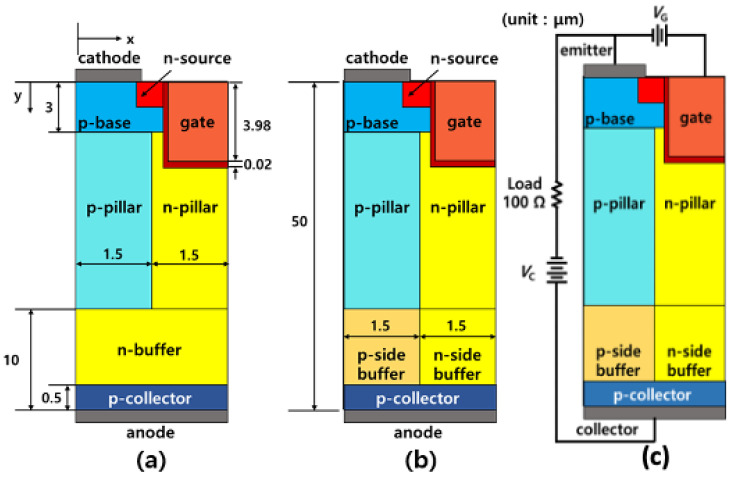
Structure of (**a**) C-SJBT and (**b**) proposed SJBT with separated n-buffer layers (SB-SJBT). (**c**) Circuit diagram for measuring turn-off loss.

**Figure 2:**
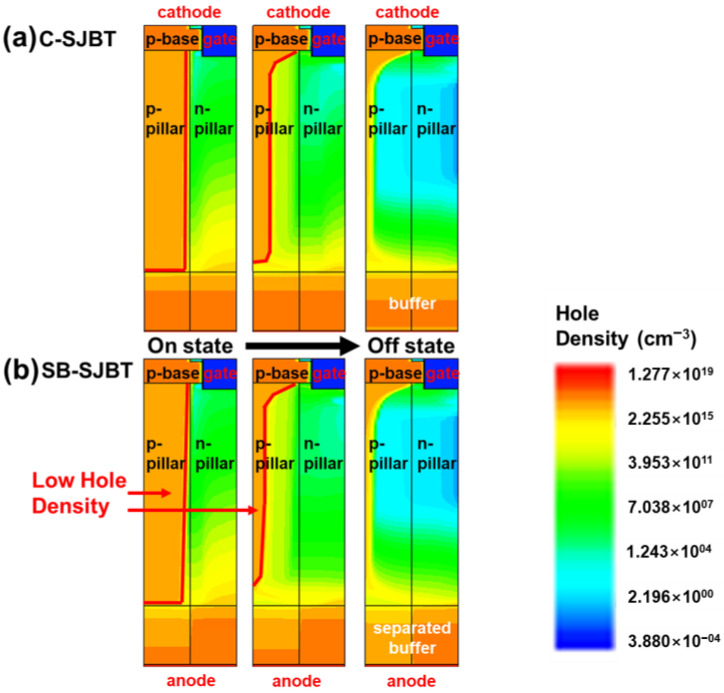
Comparison of hole density between (**a**) C-SJBT and (**b**) SB-SJBT by state. Fewer holes in SB-SJBT are injected into the p-pillar than in C-SJBT, and extraction is relatively fast.

The structures of the conventional SJBT (C-SJBT) and SB-SJBT are illustrated in Figure 1. To identify the difference in electrical characteristics between the C-SJBT and SB-SJBT, we simulated the electrical characteristics of the devices using the Synopsys Sentaurus TCAD tool. In the simulation, we designed structures using the parameters listed in Table 1. The physical dimensions of SB-SJBT were the same as those of the C-SJBT. The only difference between the C-SJBT and SB-SJBT is the adoption of a separated n-buffer layer. The separated buffer layer was composed of a p/n-side n-buffer layer that was in contact with each p/n-pillar. Because of the high n-doping concentration in the SB-SJBT, the presence of the p-buffer layer becomes a barrier preventing hole injection in the p-collector layer. Figure 2 also shows the difference in hole injection between the SB-SJBT and C-SJBT. Under the same conditions, except for the concentration of the n-buffer, fewer holes enter through the highly doped buffer. The maximum minority carrier times for the hole and electron (*t*_p_, *t*_n_) were set to 1 × 10^−5^ s and 1.5 × 10^−5^ s, the default values of TCAD [18].

**Figure 3:**
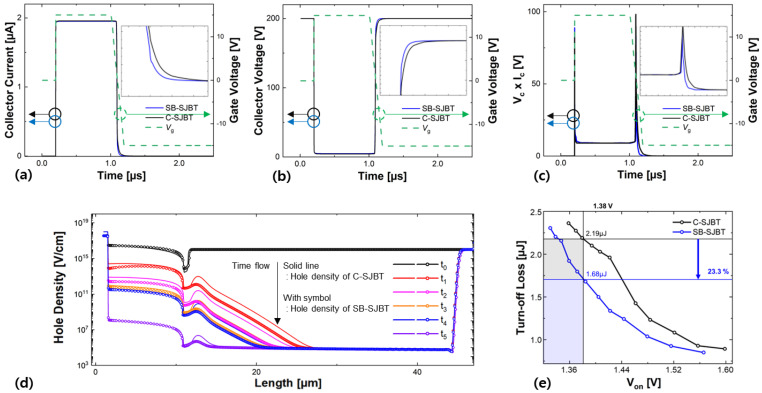
(**a**) The method for calculating the turn-off loss: the turn-off waveform of the collector current; (**b**) the collector voltage; and (**c**) power dissipation. (**d**) The graph shows the process of hole extraction over time after the C-SJBT and SB-SJBT are turned off. (**e**) The trade-off was improved by about 23.3% based on a *V*_on_ of 1.38 V.

The p-buffer layer, which has a higher doping concentration than the n-buffer layer, increases the recombination rate of holes and reduces the number of holes reaching the p-pillar. As a result, the absolute number of carriers present in the p-pillar is reduced, which results in a lower *E*_off_ than that of the conventional SJBT. Figure 3 shows an electrical characteristic curve. The gate voltage, which changes from 0 V → 15 V → −15 V over a short time, is applied to the C-SJBT and SB-SJBT (Figure 1c). It can be seen from the waveform of the collector current (Figure 3a) and collector voltage (Figure 3b) that the turn-off speed of the proposed SB-SJBT is faster than that of the C-SJBT. Figure 3d shows the distribution of holes in the device over time. Depending on the time, the hole is extracted, and t0 to t5 are indicated, which are 0.7 × 10^−6^ s, 0.9 × 10^−6^ s, 1.2 × 10^−6^ s, 1.4 × 10^−6^ s, 1.8 × 10^−6^ s, and 2.0 × 10^−6^ s, respectively. At turn-off (t_0_), both devices have almost the same hole distribution, but the figure after t_1_ to t_5_ shows that the lines with symbols (SB-SJBT) typically have a lower hole distribution than the solid line (C-SJBT). This means that the SB-SJBT extracts the holes in the p-pillar faster than the C-SJBT in the same period of time. Therefore, the proposed structure reduces *E*_off_ and improves the switching characteristics.

To quantitatively measure the degree of improvement in *E*_off_, the *V*_on_ of the C-SJBT and that of the SB-SJBT were set to similar values. The appropriate buffer concentrations for similar *V*_on_ are $9\times 10^{16}$ cm^−3^ for the C-SJBT, $3\times 10^{17}$ cm^−3^ for the p-buffer in the SB-SJBT, and $3\times 10^{16}$ cm^−3^ for the n-buffer in the SB-SJBT. *E*_off_ was defined as the integral of the product of the voltage and current (*P*_c_) from the time corresponding to 10% of the current to the time corresponding to 10% of the voltage. Therefore, the area under the power curve in Figure 3c becomes the *E*_off_ of that structure.

As a result of calculating *E*_off_ in the manner described in the previous sentence, the SB-SJBT and C-SJBT had losses of 1.68 μJ and 2.19 μJ, respectively. The turn-off characteristics of the SB-SJBT were improved by about 23.3% compared to those of the C-SJBT. The *V*_on_ was 1.38 V in both structures, and BV was 621.5 V and 626.2 V, respectively, meaning that variable control performed well. Figure 3e shows the *E*_off_-*V*_on_ trade-off for each device according to the doping concentration of the p-collector. Compared with the C-SJBT, the SB-SJBT shows an overall improved trade-off.

### 2.2. Designing ML Algorithm

**Figure 4:**
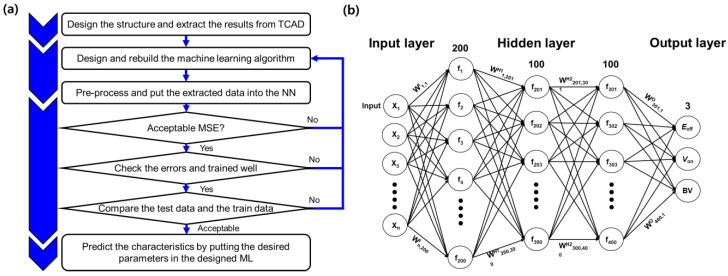
(**a**) The flow chart showing how to train the ML model and configuration of NN. (**b**) The model has 3 hidden layers (200-100-100) and extracts 3 output (*E*_off_, *V*_on_, BV) variables.

The improvement of the SB-SJBT could be verified by TCAD simulations. However, optimization with restricted computing resources cannot be performed easily or clearly. Therefore, we suggest an easy and efficient method: the ML approach. The neural network (NN) generated by the ML model provides reliable predictions through functional relationships between inputs and outputs. The ML algorithm training procedure is shown in Figure 4. To design a useful ML algorithm, the following four steps can be considered.

Step 1: Extract the examples to be used for training.

Step 2: Design the ML algorithm and train data.

Step 3: Extract the training data amount using the ML approach and determine whether it ensures a level of accuracy equal to that of the TCAD simulation.

Step 4: Redesign or complete the neural network and use it appropriately.

In the preprocessing process before training the data in Step 2, all of the data are divided into an 8:1:1 ratio for training data, test data, and validation data. Figure 4 also represents the fundamental NN of the algorithm. The following equation, expressed through weight matrices (W^I^, W^H1^, W^H2^, W^O^), bias vectors (b^1^, b^2^, b^3^, b^4^), and the activation function (φ), extracts the output (Y).

(1) $$\;Y=W^{O}\times \varphi \left(W^{H2}\times \varphi \left(W^{H1}\times \varphi \left(W^{I}\times X+b^{1}\;\right)+b^{2}\right)+b^{3}\right)+b^{4}$$

In the ML algorithm, the rectified linear unit (ReLU) activation [19] function was used for effective learning. ReLU activation is an activation function defined as the positive part of its argument. Thousands of data points were used, and the layer density was set to 200→100→100→3 with ReLU. The adaptive moment estimation (ADAM) optimizer [20,21] was used for accurate error correction. ADAM is generally used to update different values of parameters, such as AdAdagrad, Adadelta, and RMSprop, and has the advantage that the step size is not effective in rescaling the gradient. In order to obtain more precise results while compiling, the learning rate (LR) was set to 0.001. Normalization [22] by the min-max scaler in sklearn was also needed on account of the wide range of parameters. Finally, we controlled overfitting with the dropout and early stopping system of keras. Dropout is a regularization method that approximates training a large number of neural networks with different architectures in parallel. So, for this setting, 10% of the layer output is randomly ignored. Additionally, early stopping is a form of regularization when training a learner with an iterative method. The patience, which is the number of iterations the algorithm can execute before starting to overfit, was set to 10. Additionally, since the epoch at the end is a value that includes all 10 epochs when there was no significant change, the ‘best model’ was saved by subtracting 10 epochs for optimization.

## 3. Model Validation and Results

### 3.1. Verification of Model Reliability

**Figure 5:**
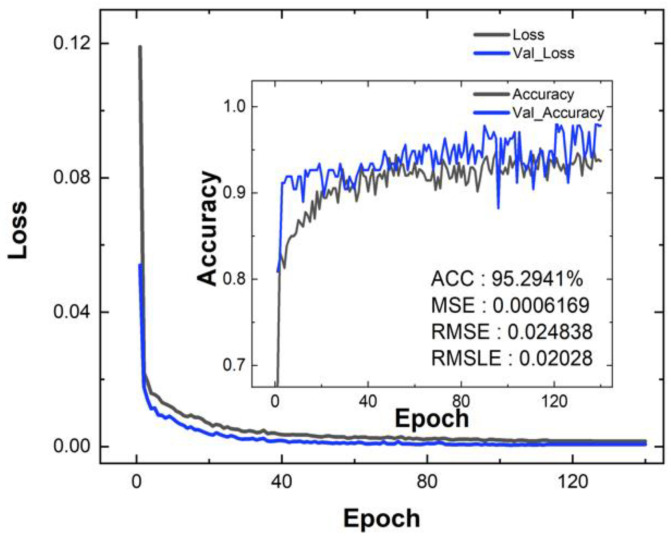
The loss approaches 0 as the epoch increases, and the accuracy according to the epoch exceeds 0.9.

**Figure 6:**
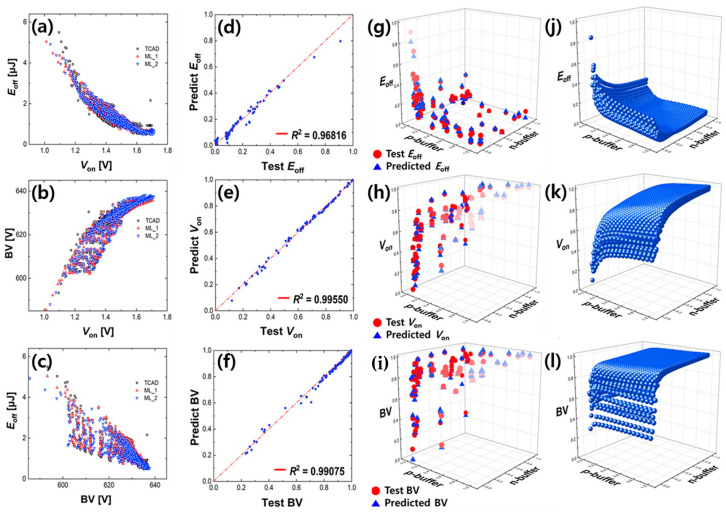
Test data set from TCAD versus data set predicted by ML model. There are (**a**) *E*_off_-*V*_on_, (**b**) BV-*V*_on_, and (**c**) *E*_off_-BV. Note that black-colored circles, red-colored triangles, and blue-colored reverse triangles indicate TCAD sample, ML sample 1, and ML sample 2, respectively. *E*_off_ (**g**), *V*_on_ (**h**), and BV (**i**) according to doping concentration are plotted in 3D, and errors are shown in (**d**–**f**), respectively. Each label is min-max-scaled, and the ideal criterion is set as the red diagonal line. *E*_off_ (**j**), *V*_on_ (**k**), and BV (**l**), the results of ML models tested with multiple samples, inherit the trends of *E*_off_ (**g**), *V*_on_ (**h**), and BV (**i**) that were approximately expected as points.

The accuracy with the MSE, RMSE, RMSLE, and Loss is shown in Figure 5. The loss and accuracy are the test data, and Val_Loss and Val_Accuracy are the training data. The Mean Squared Error (MSE) was used to estimate accuracy. Supplementally, the root mean square error (RMSE) and root mean squared logarithmic error (RMSLE) were also used. MSE is sensitive to outliers because the error is just squared. Because the square root of the MSE is RMSE, the RMSE has a unit similar to the actual result, making it easy to interpret. The RMSLE, an abbreviation of log-scaled RMSE, is robust against outliers and measures the relative error because it uses a log scale. This has a feature that imposes a large penalty for underestimation. The lower the MSE, RMSE, and RMSLE, the better the trained model. However, when it converges to zero, that is, when the accuracy approaches 100%, the model only processes the input test data as they are and cannot ignore out-of-trend data. So, it has no value as a predictor. In that sense, these results, with values of around 95%, 0.0006, 0.024, and 0.020, are reasonable. Since the RMSLE is about 0.007 lower than the RMSE, it is expected that there are some large errors, but it is not a cause for concern because the RMSE is small enough to handle. In order to check the reliability visually, the distribution plots are shown in 3D in Figure 6g–i. The doping concentration of the n-buffer below the p-pillar is placed on the *x*-axis (p_side doping), the doping concentration of the n-buffer below the n-pillar (n_side doping) is placed on the *y*-axis, and *E*_off_, *V*_on_, and BV are placed on the *z*-axis. The *E*_off_, *V*_on_, and BV extracted by TCAD are indicated by red dots, and predictions by NN are indicated by blue gradation triangles according to the *z*-axis value. One method of using NN to represent the error rate is also shown in Figure 6d–f. The closer the values are to the diagonal on the graph, the smaller the error. Numerical analysis is performed by monitoring the overall output characteristics of the structure using the coefficient of determination (*R*^2^) in the regression analysis. *R*^2^ (0 < *R*^2^ < 1) denotes the strength of the linear correlation between TCAD data and the predicted results. The calculation of the *R*^2^ value is as follows:

(2) $$SS_{\mathrm{total}}=\sum {\left(y_{i}-\bar{y}\right)}^{2}$$

(3) $$SS_{\mathrm{regression}}=\sum {\left(y_{i}-y_{\mathrm{regression}}\right)}^{2}$$

(4) $$R^{2}=1-\frac{SS_{\mathrm{regression}}}{SS_{\mathrm{total}}}$$

where $SS_{\mathrm{total}}$ and $SS_{\mathrm{regression}}$ are the squared total error and regression error, respectively, and *y*_i_, $\bar{y}$, and *y*_regression_ are each data point, the mean value, and the regression value, respectively. As a result, the *R*^2^ values of *E*_off_, *V*_on_, and BV are 0.96816, 0.99550, and 0.99075, respectively. Since the value of *R*^2^ is close to 1, it can be said that the model explains the data well. The numerical analysis is also shown in Table 2. The accuracy of the prediction data was evaluated once more using the confidence interval calculated by the standard error of the mean and the standard deviation [23].

(5) $$\mathrm{Standard}\;\mathrm{error}\;\mathrm{of}\;\mathrm{mean}=\frac{\sigma }{\sqrt{n}}$$

(6) $$\mathrm{Standard}\;\mathrm{error}\;\mathrm{of}\;\mathrm{deviation}\;\approx \;\frac{\sigma }{\sqrt{2\left(n-1\right)}}$$

*µ*: mean;
*σ*: standard deviation;
*n*: number of samples in one set of data.

**Figure 7:**
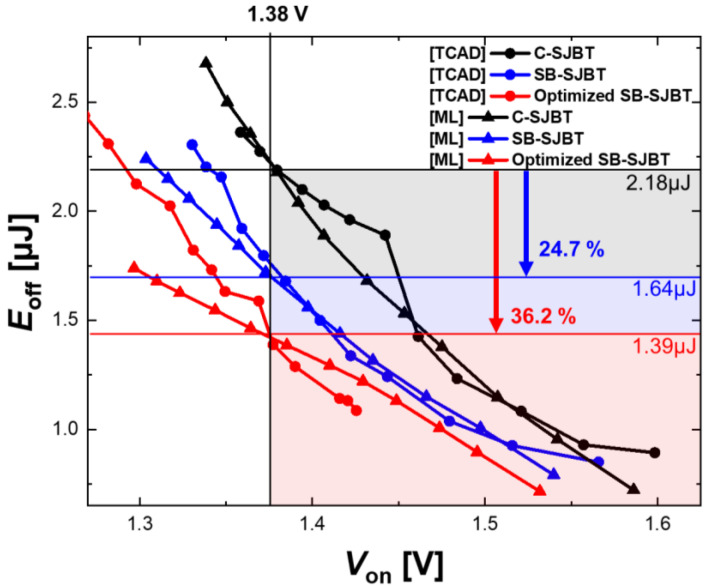
Comparing the trade-off graph extracted by the TCAD tool with the graph predicted by NN shows very high similarity. In addition, the optimal structure extracted by ML shows an improved trade-off.

The trained model does not simply mimic TCAD but corrects for some large errors that do not fit the trend. It makes continuous trends more reasonably predictable and helps to increase the reliability of the results. Indeed, the σ of ML is observed to be smaller than that of TCAD. The blue spheres in Figure 6j–l including the blue triangles in Figure 6g–i are the results of running ML with 120,000 input clusters not simulated by TCAD and show the complemented overall trend of the red dots (simulation) in Figure 6g–i. A more detailed example is shown in Figure 7. The superior characteristics of the SB-SJBT in terms of the trade-off compared to those of the C-SJBT were learned well as is. On the other hand, the analysis via ML more clearly demonstrates the advantages of the SB-SJBT than the analysis via TCAD. This is because, unlike TCAD, ML infers more reasonable results while adjusting for large outliers from calculations.

### 3.2. Optimization

With the learned NN model, we could expect the proposed SB-SJBT to have an *E*_off_ improvement of 24.7% at *V*_on_ 1.38 V. However, this structure is not guaranteed to have an optimal trade-off. A combination of optimized buffer concentrations and characteristics can be extracted by ML [24,25,26]. The red triangles in Figure 7 are characteristic of a structure with an optimized trade-off characteristic having a combination of buffer concentrations of $3\times 10^{16}$ cm^−3^ and $1\times 10^{18}$ cm^−3^, respectively. Based on the same *V*_on_ of 1.38 V, the *E*_off_ of the optimized SJBT improved by 36.2% (from 2.18 μJ to 1.39 μJ) for the C-SJBT, which is superior to the SB-SJBT, which improved by 24.7% to 1.64 μJ. In addition to this advantage, the ML approach has shown overwhelming advantages in terms of time during optimization. In this experiment, the duration was improved by more than 118% compared to the TCAD simulation, which took more than 10 min to compute a node, even considering the extraction time for approximately 1000 data points, the model training time, and the operation time required for ML. Moreover, it can be seen that the trade-off is also improved in TCAD when executed as a parameter in ML.

### 3.3. Reverse Engineering by NN

Typical simulations such as TCAD can only check discrete characteristics that are determined by specific input parameters. However, the analysis using NN was shown to have a tremendous advantage in that it generates a function that predicts the fluid point from the relationship between the input data and the output data. This makes reverse engineering possible. In addition, there are countless combinations with similar characteristics, so it is difficult to find each one, and compared to directly adjusting the input parameters by intuition, the method using NN is more effective in obtaining a precisely optimized doping level and checking the output characteristic. To give an example, by adopting the characteristics previously identified as structural characteristics of the SB-SJBT (*E*_off_ = 1.64 μJ, *V*_on_ = 1.38 V), structures with similar characteristics were extracted and are listed in Table 3. It turns out that structure B (SB-SJBT) has the same characteristics as expected. Structures A, C, and D were also found with almost the same characteristics, and as shown from the characteristics of structures A, C, and D, it can be seen that *E*_off_ and BV decrease as the p-buffer concentration increases, and *V*_on_ is compensated as the n-buffer concentration decreases. As such, the ML approach has the advantage of being able to target specific characteristics and flexibly control other parameters.

## 4. Conclusions

The SB-SJBT is proposed for the purpose of reducing *E*_off_, which is considered a major problem of the SJBT, and it needs to be optimized. In general, TCAD simulation is regarded as the most powerful means of semiconductor analysis for device design. However, only the characteristics of a specific parameter can be identified by TCAD simulation. This makes it inefficient in terms of the time required for designing and optimizing the structure or considering additional variables (e.g., process conditions). Therefore, an ML model is proposed. The ML approach ensured a remarkably short time with reliable accuracy in this study. Specifically, the *R*^2^ of *V*_on_ and *E*_off_ reached 0.995 and 0.968, respectively, and the structure could be optimized to have an *E*_off_ improvement of 36.2% over the C-SJBT based on a *V*_on_ of 1.38 V. Moreover, the required input parameters could be easily obtained by using target output characteristics as necessary. Although there is still a simple functional relationship between the input and output, as the technology develops, there are endless prospects, such as predicting the carrier’s movement path or the vector diagram of the electric field. In addition, the proposed ML utilization method is expected to be very strategic for various database applications (e.g., finding the optimal equipment and conditions for a unit process), especially in a power semiconductor analysis where the transistor size is comparatively big and requires lots of computation.

## Figures and Tables

**Table 1:** Device parameters for the simulation.

Parameter	C-SJBT	SB-SJBT
Width	3.0 µm	3.0 µm
Length	50.0 µm	50.0 µm
Gate depth	5.0 µm	5.0 µm
p/n-pillar width	1.5 µm	1.5 µm
p/n-side n-buffer width	-	1.5 µm
n-buffer layer depth	9.5 µm	9.5 µm
p-base doping	$6\times 10^{16}$ cm^−3^	$6\times 10^{16}$ cm^−3^
n-source doping	$5\times 10^{20}$ cm^−3^	$5\times 10^{20}$ cm^−3^
p/n-pillar doping	$1\times 10^{16}$ cm^−3^	$1\times 10^{16}$ cm^−3^
n-buffer doping	$9\times 10^{16}$ cm^−3^	-
p-collector doping	$1\times 10^{18}$ cm^−3^	$1\times 10^{18}$ cm^−3^

**Table 2:** Mean and standard deviation of the performance metrics.

Data Set		*E*_off_ [µJ]		*V*_on_ [V]		BV [V]	
		µ	σ	µ	σ	µ	σ
TCAD		1.19	6.94	1.511	0.137	629.03	8.719
ML results 1		1.18	6.62	1.509	0.138	628.57	8.780
ML results 2		1.16	6.63	1.513	0.137	629.15	8.979
Confidence interval	Max	1.24	7.27	1.571	0.142	629.63	10.489
	Min	1.14	6.60	1.451	0.132	619.99	8.295

**Table 3:** Similar structures based on the set output.

Structure	A	B	C	D
p-buffer doping [cm^−3^]	$2.9\times 10^{17}$	$3\times 10^{17}$	$3.1\times 10^{17}$	$3.4\times 10^{17}$
n-buffer doping [cm^−3^]	$4\times 10^{16}$	$3\times 10^{16}$	$2\times 10^{16}$	$2\times 10^{16}$
*E*_off_ [μJ]	1.73	1.64	1.56	1.53
*V*_on_ [V]	1.39	1.38	1.37	1.38
BV [V]	622.4	620.5	617.2	617.1

## Data Availability

No new data were created or analyzed in this study. Data sharing is not applicable to this article.

## References

[1] Aghdam M.G.H., Thiringer T. Comparison of SiC and Si Power Semiconductor Devices to Be Used in 2.5 Kw Dc/Dc Converter. Proceedings of the International Conference on Power Electronics and Drive Systems.

[2] Shimizu H., Harada J., Bland C. Role of Optimized Vehicle Design and Power Semiconductor Devices to Improve the Performance of an Electric Vehicle. Proceedings of the IEEE International Symposium on Power Semiconductor Devices & ICs (ISPSD).

[3] Wang Y., Li Y., Dai X., Zhu S., Jones S., Liu G. Thermal Design of a Dual Sided Cooled Power Semiconductor Module for Hybrid and Electric Vehicles. Proceedings of the Applied Power Electronics Conference and Exposition—APEC.

[4] Bauer F. The MOS Controlled Super Junction Transistor (SJBT): A New, Highly Efficient, High Power Semiconductor Device for Medium to High Voltage Applications. Proceedings of the IEEE International Symposium on Power Semiconductor Devices and ICs (ISPSD).

[5] Bauer F.D. (2004). The Super Junction Bipolar Transistor: A New Silicon Power Device Concept for Ultra Low Loss Switching Applications at Medium to High Voltages. Solid. State. Electron..

[6] Antoniou M., Udrea F. Simulated Superior Performance of Superjuction Bipolar Transistors. Proceedings of the International Semiconductor Conference, CAS.

[7] Laska T., Münzer M., Pfirsch F., Schaeffer C., Schmidt T. The Field Stop IGBT (FS IGBT)—A New Power Device Concept with a Great Improvement Potential. Proceedings of the IEEE International Symposium on Power Semiconductor Devices and ICs (ISPSD).

[8] Eicher S., Bauer F., Weber A., Zeller H.R., Fichtner W. Punchthrough Type GTO with Buffer Layer and Homogeneous Low Efficiency Anode Structure. Proceedings of the IEEE International Symposium on Power Semiconductor Devices & ICs (ISPSD).

[9] Eicher S., Bauer F., Zeller H.R., Weber A., Fichtner W. Design Considerations for a 7kV/3kA GTO with Transparent Anode and Buffer Layer. Proceedings of the PESC Record—IEEE Annual Power Electronics Specialists Conference.

[10] Widjaja I., Kurnia A., Divan D., Shenai K. Conductivity Modulation Lag During IGBT Turn On in Resonant Converter Applications. Proceedings of the 52nd Annual Device Research Conference.

[11] Shenoy P.M., Bhalla A., Dolny G.M. Analysis of the Effect of Charge Imbalance on the Static and Dynamic Characteristics of the Super Junction MOSFET. Proceedings of the IEEE International Symposium on Power Semiconductor Devices and ICs (ISPSD).

[12] Yamashita J., Yamada T., Uchida S., Yamaguchi H., Ishizawa S. Relation between Dynamic Saturation Characteristics and Tail Current of Non-Punchthrough IGBT. Proceedings of the Conference Record—IAS Annual Meeting (IEEE Industry Applications Society).

[13] Suwa T., Hayase S. Investigation of Tcad Calibration for Saturation and Tail Current of 6.5kv Igbts. Proceedings of the International Conference on Simulation of Semiconductor Processes and Devices, SISPAD.

[14] Wei J., Zhang M., Chen K.J. Design of Dual-Gate Superjunction IGBT towards Fully Conductivity-Modulated Bipolar Conduction and Near-Unipolar Turn-Off. Proceedings of the International Symposium on Power Semiconductor Devices and ICs.

[15] Luo X., Zhang S., Wei J., Yang Y., Su W., Fan D., Li C., Li Z., Zhang B. A Low Loss and On-State Voltage Superjunction IGBT with Depletion Trench. Proceedings of the International Symposium on Power Semiconductor Devices and ICs.

[16] Noh J.S., Lee K., Park S.H., Jeon M.G., Yoon T.Y., Kim J.H. Improvement in Turn-off Loss Characteristic for The Super Junction IGBT with Separated n-Buffer Layers. Proceedings of the Nano Korea.

[17] Li W., Wang B., Liu J., Zhang G., Wang J. (2020). IGBT Aging Monitoring and Remaining Lifetime Prediction Based on Long Short-Term Memory (LSTM) Networks. Microelectron. Reliab..

[18] Tyagi M.S., Van Overstraeten R. (1983). Minority Carrier Recombination in Heavily-Doped Silicon. Solid. State. Electron..

[19] Agarap A.F. (2018). Deep Learning Using Rectified Linear Units (Relu). arXiv.

[20] Kingma D.P., Ba J.L. Adam: A Method for Stochastic Optimization. Proceedings of the 3rd International Conference for Learning Representations.

[21] Zeiler M.D. (2012). ADADELTA: An Adaptive Learning Rate Method. arXiv.

[22] Ba J.L., Kiros J.R., Hinton G.E. (2016). Layer Normalization. arXiv.

[23] Ahn S., Fessler J.A. (2003). Standard Errors of Mean, Variance, and Standard Deviation Estimators.

[24] Ko K., Lee J.K., Kang M., Jeon J., Shin H. (2019). Prediction of Process Variation Effect for Ultrascaled GAA Vertical FET Devices Using a Machine Learning Approach. IEEE Trans. Electron Devices.

[25] Lim J., Shin C. (2020). Machine Learning (ML)-Based Model to Characterize the Line Edge Roughness (LER)-Induced Random Variation in FinFET. IEEE Access.

[26] Mehta K., Raju S.S., Xiao M., Wang B., Zhang Y., Wong H.Y. (2020). Improvement of TCAD Augmented Machine Learning Using Autoencoder for Semiconductor Variation Identification and Inverse Design. IEEE Access.

